# Does gender and education of the households’ heads matter for wealth accumulation in Vietnam? Evidence from a recent decade^☆^

**DOI:** 10.1016/j.heliyon.2023.e22836

**Published:** 2023-11-25

**Authors:** Duc Hong Vo, Anh The Vo, Chi Minh Ho

**Affiliations:** The Research Centre in Business, Economics and Resources, Ho Chi Minh City Open University, 97 Vo Van Tan Street, District 3, Ho Chi Minh City, Viet Nam

**Keywords:** Education, Gender, Wealth, Vietnamese households, VHLSS

## Abstract

Asians believe that education plays a crucial role in earning higher income for individuals and accumulating larger wealth for households. Educational achievements have been generally considered an indicator of success. However, gender bias in favour of males as the household heads still exists in Asian societies due to the significant influence of Confucian belief. This study investigates the independent effect of education and gender of the households' heads and their joint effect on household wealth in the Vietnamese context in the most recent decade using data from the Vietnam Household Living Standards Surveys (VHLSSs) between 2010 and 2020. Our study uses quantile regression and propensity score matching methods to ensure the robustness of the empirical results. We find that the education of the household heads contributed to increased household wealth in Vietnam in 2010 and 2020. However, household wealth decreases across different wealth quantiles when the households' heads are females. These findings confirm the currently deep-rooted gender stereotypes and a ‘gendered structure’ economy in Vietnam and require attention from the Vietnamese government. In addition, our empirical results reveal that being a female as the household head with a degree/certificate from a general education will increase household wealth. Our empirical results have largely been consistent across different wealth distributions.

## 1 Introduction

Vietnam has made remarkable educational strides after the economic reform initiated by the nation's *Doi Moi* (*Economic Reform)* policy in 1986, shifting the economy to a market-oriented economy. The governments have implemented policies to encourage private enterprises and attract foreign investment, supporting economic growth and job opportunities, and this period exhibited improvements in living standards. However, income inequality also increased [1,2]. During the 1990s, Vietnam had achieved rapid economic growth. Poverty rates have declined, and social indicators, such as access to education and healthcare, have been improved [3]. After one decade, Vietnam has further integrated into the global economy, becoming a World Trade Organization (WTO) member in 2007.

Besides these economic achievements after the “*Doi Moi*” (Economic Reform) in 1986, educational attainment is the ultimate result of prioritizing education in the national strategies. The Vietnamese government has emphasized education for young and out-of-school people to pursue inclusive education. In 1992, the “Education for All” (EFA) 1993 to 2000 Action Plan was implemented [3]. The core of the first (1993–2000) EFA plan aims to achieve universal primary education with high quality, close the gap in gender inequality at every educational level, and provide sufficient basic education and training out-of-school people to increase literacy rate. The 2003–15 EFA plan set five primary objectives: transitioning from quantity to quality, achieving universal primary and lower secondary education, offering lifelong learning opportunities, encouraging full participation in education, and improving resource allocation [4]. [5] reveal that Vietnam has made significant achievements in increasing school enrollment across all levels over the past two decades, achieving near-universal primary and lower secondary enrollment. For upper secondary education, remarkable growth in the enrollment rate is witnessed. In the early 1990s, only one in four children were enrolled at the upper secondary level. However, this figure had risen to almost three out of four children by 2006. Despite the virtually universal enrollment rate, the report by Vietnam's Ministry of Education and Training (MOET) and UNICEF Vietnam (2021) shows that the quality of Vietnamese primary education outperforms the average of five other ASEAN countries, including Cambodia, Laos, Myanmar, Malaysia, and Thailand. In terms of university education, several Vietnamese universities are listed in quality world university rankings such as the Academic Ranking of World Universities (ARWU), Times Higher Education (THE), and the Quacquarelli Symonds (QS) ranking for the first time in 2019. Moreover [6], report on “Growing Smarter: Learning and Equitable Development in East Asia and Pacific" considers Vietnam one of the world's top ten performing education systems. Also, the Human Capital Index report by Ref. [7] reveals that Vietnam ranks 15th in terms of educational outcomes, on par with countries such as the Netherlands, New Zealand, and Sweden. Another noteworthy aspect of the Vietnamese education system is the performance of its students on international assessments and competitions. For example, in the Programme for International Student Assessment (PISA) conducted in 2012, 2015 and 2018, Vietnam's scores surpassed the average of the OECD and several prominent countries such as the United States and the United Kingdom. Regarding international competitions (e.g., International Mathematics Olympiad, International Physics Olympiad, International Chemistry Olympiad, International Biology Olympiad), Vietnam stands in a high-ranking position worldwide [8].

Notwithstanding the increased literacy rates and primary education coverage, substantial educational disparities persist among rural and urban regions and ethnic communities. Primary students in remote areas and ethnic minorities show much lower abilities in mathematics, reading and writing [9]. There is a significant difference regarding the level of education and the attendance rate between urban and rural regions [10]. However, no evidence is found for schooling attendance rate by gender at primary and secondary school. However, when it comes to high school education, there is a noticeable discrepancy in the overall attendance rate, with boys exhibiting a 7.1% lower enrollment than girls. Additionally, while the primary net enrollment rate of the ethnic minority groups has virtually converged with the ethnic majority groups, disparities significantly persist at the lower secondary and secondary enrolment rates [5]. At the higher education level, the recent expansion has resulted in an imbalance between disciplines, intense conflicts between scale, quality, and effectiveness, unalignment between education and the demand for human resources, and failure to meet production requirements, leading to a significant waste for society [11].

Vietnam's Provincial Governance and Public Administration Performance Index (PAPI) survey highlights poverty as a pressing concern that requires immediate attention from the government. Following Vietnam's "Doi Moi" in 1986, the government pledged to adopt a market-oriented economy while still conserving fundamental characteristics of socialism. However, the National Center for Social Sciences and Humanities (NCSSH) confirms a widening wealth disparity among Vietnamese. Specifically, an earnings difference of 10 times is found between the population's top 20% and the bottom 20%. By 2020, due to the impact of the COVID-19 pandemic and the effectiveness of social welfare policies targeting the poor and policy-beneficiary households, the low-income group has increased, and the income disparity has tended to slightly decrease to a ratio of 8.1 times [10]. Nevertheless, this ratio is still relatively high. In addition, the General Statistics Office (GSO) report in 2021 confirms that gender inequality still significantly exists in access to employment opportunities, employment status, education, and training. The labour market inequalities are even aggravated by the COVID-19 pandemic, which caused a decrease in working hours and increased living rates among women [12]. As a result, gender inequality in wealth and income can be negatively affected because the amount they can directly allocate towards savings and investments and contributions to wealth based on earnings will decrease. The United Nations Development Programme (UNDP) in Vietnam has expressed concerns regarding the growing wealth gap and its potential implications for policymakers. It is believed that such inequality could hinder job opportunities and access to essential services, particularly in regions such as the Northern mountainous areas and the central highlands. The UNDP Vietnam has also identified income equality as a major national issue.

In the Vietnamese context, women comprise a substantial portion of the education sector. They constitute a significant majority in the teaching profession, with almost 100% in preschool education and around 70% in primary and secondary education [13]. In addition, about 37% of female teachers at all levels have college degrees. Moreover, the Department of Education and Training at the district level has more than 90% of women participating in leadership positions [14]. These successes in tackling gender inequality in education have laid the foundation for the Vietnamese Government to promulgate the national strategy on gender equality for the period 2021–2030. This strategy contains ambitious goals in the education and training sector, such as: (i) the rate of newly enrolled female students in the vocational education system will reach over 30% by 2025 and 40% by 2030; (ii) the proportion of female teachers with the master's degrees will reach no less than 50% from 2025 onwards; and (iii) Female PhDs will reach 30% of all doctorate degree holders by 2025 and 35% by 2030 [15]. This strategy demonstrates the government's exhaustive commitment and efforts and the strong will of Vietnamese females to bridge the gap between genders in education.

Regarding the gender wealth gap, recent research from Ref. [16] confirms a gender wealth gap between male and female-headed households in Vietnam. These authors consider that the differences in wealth between household head's gender (i.e., the household headship) might be affected by various factors. First and foremost, the national ideology represents the dominant effect on social perception of gender preference (e.g., Confucianism favours men over women). Besides, female headship is usually defined as the husband being absent or unable to make household decisions among coupled households. However, female headship is also determined when a woman owns a large share of household resources.

Our paper investigates the independent effect of education and gender of the households' heads and their joint effect on household wealth in Vietnam in the most recent decade between 2010 and 2020. Various attempts have been made to examine the impact of educational attainment on wealth accumulation [[16], [17], [18], [19], [20], [21], [22]] in the existing literature. However, our study is one of the first of its kind, focusing on the joint effect of education and gender of the households' heads on wealth accumulation at the household level in Vietnam in the most recent decade between 2010 and 2020.

We argue that education may improve wealth accumulation in Vietnam. However, continuing gender bias in favour of males and boys has led Vietnamese society to a suboptimal structure of the households, leading to reduced wealth accumulation for the household. In addition, we investigate the possibility that females as the households' heads with education degrees/certificates will support or hinder wealth accumulation at Vietnam's household levels. Findings from our study provide insightful implications for practitioners and policymakers in formulating and implementing appropriate socioeconomic policies.

Following this introduction, we review the relevant literature in section 2. Data and research methods are discussed in section 3. Key findings are presented and discussed in section 4. Section 5 concludes and provides policy implications.

## 2 Literature review

A household's wealth refers to its physical assets, such as property and other fixed assets, and its liquid financial assets, such as savings accounts, equities, and bonds [23]. Wealth is formed from the process of resource accumulation over time. However, this process is impacted by physical resources and various personal, institutional, and family-related factors [24]. In general, larger economic resources result in better living conditions, more consumption, financial independence, and a lower likelihood of depending on social security. Through intergenerational transfers, wealth may protect people from income losses, influence power relationships, and change social mobility [25]. Depending on its elements, wealth may provide utility through the direct use of resources such as houses and vehicles, income through the interest from savings, and security through the protective function of accumulated savings [26]. Empirically, several studies have examined the impact of the household head's characteristics on wealth accumulation [18,[27], [28], [29]]. Particularly [[28], [29], [30], [31]], confirm the importance of saving behaviour. Savings and wealth accumulation are affected by income, interest rates, and risk attitudes.

Another factor influencing household wealth accumulation is the educational level of the household head [[29], [30], [31]]. Previous research confirms the return of education to individual income [[32], [33], [34]]. Specifically, individuals with higher education have higher incomes, lower unemployment chances, and more prestigious jobs than those with lower education. Furthermore, at the aggregate level [33,34], found that education enhances economic growth and reduces income inequality. Recent studies argue that educational level improves household wealth accumulation [17,18,35]. Education improves individuals' numeracy skills, resulting in better allocation of household financial resources, leading to a more efficient wealth accumulation.

The household head is generally considered a decision-maker for the family's well-being, specifically related to the household's financial allocation, including spending and saving. Furthermore, the head of the family is responsible for dealing with government authorities. This responsibility aligns with [36] description of the household's head as "the person who represents the household in dealing with the larger community". As a result, to comprehend family wealth accumulation, a significant factor that has to be included in the analysis is the head's characteristics and other factors such as household characteristics, regional specifications, and many others.

Headship is generally simple in families with only one adult. Female-headed households (FHHs) denote families with females as the households' heads. Male-headed households (MHHs) are famines headed by a man. Previous studies have demonstrated that single male-head households accumulate wealth similar to typical married couples [37,38]. However [39], observe that single male-headed families and their counterparts with at least one child achieved between 5% and 15% lower wealth accumulation than married households. Besides [[37], [38], [39]], found that unmarried women with children achieved the lowest wealth accumulation. However, a broader set of family characteristics determines whether a household is FHH or MHH for married couples or multigenerational families. In Vietnam, men are usually assigned the role of household heads arising from the impact of traditional Confucian beliefs [40]. Furthermore, land distribution favours MHHs under the national 1986 *Doi Moi* implemented in 1986, adding a financial incentive to designate males as the household heads [41,42].

From another aspect, the Vietnamese inheritance customs have historically favoured men. It is common to leave residential land to sons rather than daughters. In addition, sons are expected to maintain the family's ancestral altars. Specifically, the first or the last son is likely to get a larger share of a parental inheritance than his siblings. Within special circumstances, parents without sons arrange inheritance rights for a nephew or male relatives instead of their daughters to keep property ownership [43]. This also implies that women who inherit significant wealth are relatively uncommon. Based on these findings from the literature, we anticipate that women and FHHs will have disadvantages when it comes to inheriting and owning assets compared to males and MHHs. Several obstacles stem from ancient Confucian inheritance practices, prohibiting women from holding major assets such as land and residential property.

However, implementing the *Doi Moi* land reforms through granting land-use certificates (LUCs) to farming families strengthens existing land ownership inequalities, benefiting MHHs. According to Ref. [44], because LUCs could only include one person and were filled out by the household head, and most household heads were male, comparatively few women were mentioned on the LUCs at first. Changes made in 2001 required the names of both husband and wife to appear on the LUCs if the land was jointly held. The new restrictions, however, were not properly enforced. Women also suffered due to age-based land distribution systems, with working-age persons receiving the largest share and young children receiving the smallest. Women are less likely to be in the working age since their legal retirement age is five years lower than men. Consequently, the quantity of land given to women between 55 and 59 was half that of males between 55 and 59 [44]. When a woman divorces, she frequently relinquishes her property rights to her husband's family. This occurs despite the Vietnamese Marriage Law specifying that land and other assets are to be shared equally between the spouses upon divorce, and divorcees have the right to seek a share of residential land acquired during the marriage in court. The practice of pursuing claims on property after divorce has yet to be prevalent, illustrating the gaps that persist between Vietnam's laws and customary practices [45]. In rural regions, the effects on women's wealth following divorce are frequently exacerbated since they are not permitted to return to their natal family, limiting their access to residential land [45].

On this aspect, gender inequality is directly related to the difference in wealth accumulation. Female-headed households (FHHs) are regarded as among the lowest of the poor [46]. FHHs endure disadvantages in many nations, including Vietnam, because of gender norms restricting women's participation in or benefits from economic activities and their limited capacity to own economic assets. Many studies have demonstrated the major danger of poverty for FHHs. In a comparative review of over sixty studies on Latin America, Africa, and Asia [47], concluded that two-thirds of the households headed by women in those countries were poorer than those led by men. Many studies, in general, discuss the hurdles to wealth-building experienced by women in Vietnam [48]. consider that women are less likely than males to acquire credit, and when they do, they often receive less than their male counterparts [49]. argue that female-led businesses are more likely to be denied credit than male-led businesses, and these barriers are especially pronounced in male-dominated industries such as food and beverage, fabricated timber, rubber and plastics, fabricated metal, and electronics. Male-led businesses account for more than half of each industry in these industries during times of tight monetary policy. These discrepancies in running a business can be explained by women's lower average level of educational attainment, larger unpaid care obligations, and overall lower access to collateral [50].

Regarding previous research on household wealth, besides the above critical problems of gender inequality [16], educational level is another substantial factor affecting household wealth. Previous research confirms the relationship between higher education and income [18,21]. [18] argue that educational level is related to outstanding numeracy and crystallized intellect. Their study supports the belief that education may improve cognitive skills, particularly numeracy, leading to large wealth creation [21]. investigate the influence of household wealth on educational demand. They found that wealth and education demand changes were substantial and positively associated.

Recently [16,17], have investigated the contribution of education to household wealth within the context of the gender wealth gap in Vietnam [17]. focus on the return of education to household wealth and discuss gender as a moderating factor. In contrast [16], particularly examines the gender wealth gap. None of the above studies investigates the joint effect of gender and education on wealth accumulation in the Vietnamese context.

On this basis, the contributions of our study to the existing literature regarding gender studies and household wealth are twofold. First, this paper extends previous studies in examining the joint effects of the gender of the household heads and their education on the households' wealth in the Vietnamese context. While the independent effects of the gender of the household heads and their education on the households' wealth have been investigated, albeit limited, their joint effects have largely been neglected. Specifically, we employ both general education and vocational training certificates to represent a level of education. Second, the quantiles regression and a propensity score matching method are used to investigate the dynamic effects of gender and education on household wealth across different distributions in Vietnam. Details of our analytical framework and econometric strategy are presented in the next section.

## 3 Data and research methodology

The current paper examines the independent effect of the gender and education of the households' heads and their joint effects on household wealth in the Vietnamese context. Furthermore, we particularly focus on the changes in these effects occurring in the recent decade between 2010 and 2020. The Vietnam Household Living Standards Survey (VHLSS) database provides sufficient data on various aspects of Vietnamese households, allowing us to conduct our analysis. The VHLSS survey has been started since 1992 and is conducted biannually by the General Statistics Office of Vietnam with support from the World Bank. The survey includes various indicators related to individual and household characteristics across 63 provinces in Vietnam.

Previous studies have investigated the effect of the household heads’ characteristics, including gender and educational level, on household wealth [16,17]. The model is expressed in equation (1) as follows:

(1) $${NW}_{i}={Head\;Education}_{i}+{Head\;Gender}_{i}+X_{i}+e_{i}$$

where ${NW}_{i}$ represents the household's net worth that measures the wealth level of a household; ${Head\;Education}_{i}$ represents the head's educational characteristics; ${Head\;Gender}_{i}$ denotes the head's gender; $X_{i}$ is a vector of control variables; and $e_{i}$ is the disturbance term.

This study extends the previous analysis by investigating the independent effect of the gender and education of the households' heads and their joint effect on the households' wealth in the Vietnamese context. We introduce the interaction term between the household heads' gender and educational achievement. As such, equation (1) above now becomes equation (2):

(2) $${lnNW}_{i}={Head\;Education}_{i}+{Head\;gender}_{i}+{Head\;gender}_{i}*{Head\;Education}_{i}+X_{i}+\mu_{i}$$

The size of a household is measured by the number of adults and children living in the household. The dependent members, or locational characteristics, are considered other household characteristics affecting the household's wealth [51]. As such, we incorporate these variables into the model to form equation (3) as follows:

(3) $${lnNW}_{i}={Head\;Education}_{i}+{Head\;gender}_{i}+{Head\;gender}_{i}*{Head\;Education}_{i}+{Household\;Characteristic}_{i}+Z_{i}+\varepsilon_{i}$$

Where: ${Household\;Characteristic}_{i}$ includes household size, geographical characteristics, ethnicity, and other factors.

The household's net worth is the net value between the total household's assets and the total debts. Data on household wealth are collected from responses to different questions. Assets and debts are recorded at the household level. As such, net worth is the difference between assets and debts. The VHLSS surveys classify the education level of respondents into (i) general education and (ii) vocational training. General education includes three levels of education (the primary, secondary, and high school levels): university or college and post-graduate studies. Vocational training includes elementary, intermediate, and advanced training.

Household size is measured by the number of household members currently living in the household. However, adults and children will have different contributions to the household's wealth. As such, we employ the equivalent scale function suggested by Ref. [52] to measure household size:

$$ES={\left(AD+\gamma CH\right)}^{\delta }$$

where: ES denotes the equivalent household size. “AD” represents the number of adults living in the household. The “CH” denotes the number of children under 15 years old living in the household. γ, ranging from 0 to 1, represents the equivalent expenditure of a child compared to an adult. Finally, δ, ranging from 0 to 1, represents the household economies of scale.

[52] consider that it is less costly to raise a child in developing countries. As such, γ can take the values from 0.3 to 0.5. The household economy of scale in these countries should be higher than 0.9 but less than 1 because total fixed expenditures have relied on necessary goods such as foods and beverages [52]. Previous research suggested γ = 0.65 and δ = 1 for Vietnam [53]. However, we use γ = 0.55 and δ = 0.9. Our view is supported by the following observations. First, Vietnam has been a lower-middle-income country since 2009 [54]; δ should be lower than 1e. Besides, the economic circumstances of the households in 2020 were better than in 1998. we use a value of 0.55 for γ.

Our analysis uses the matching method, Propensity Score Matching (PSM), to conduct a matching sample. Education is used as the treatment effect. PSM conducts a paired sample which shares relatively identical characteristics. As such, the PSM has the advantage of overcoming potential selection bias issues, which is a critical endogenous issue in the analysis using survey data. Instead of using gender as the treatment, as [16], considering education as the treatment provides insight regarding the return of education to household wealth. To focus on the effect of education on wealth, we assign educational level one of the two levels: (i) the "*with education*” and “*without education*". In other words, the household head is recorded as "with education" if they receive at least a degree in general education or a certificate from vocational training. Alternatively, the household head is recorded as "without education".

After conducting a matching sample using the PSM technique, the quantile regression allows us to estimate the effects of education, gender, and their interaction across different wealth distributions. With the quantile regression, we can observe and compare the changes in educational returns to household wealth across different levels of wealth.

Table 1 presents the descriptive statistics of our data. The number of respondents attending the VHLSS survey in 2020 is lower compared to 2010. However, an increase of one hundred FHHs occurred in 2020. This shows the rise of female participation in Vietnamese society from 24% of FHHs in 2010 to 25% in 2020. Regarding general education, the general education achievements favour the MHHs in both 2010 and 2020, as observed by a higher proportion of the head with the status “With education”. This evidence aligns with previous findings from Ref. [50]. Interestingly, the increased proportion of FHHs whose heads are achieving a general education is larger than the MHHs. Particularly, from 2010 to 2020, an additional seven per cent of the household heads with a general education diploma were found within the FHHs, and a five per cent increase was observed in the sample of MHHs. Regarding vocational training, the data reveals a reduction in attending vocational training in FHHs from 13 to 11% between 2010 and 2020. However, achieving a vocational training certificate increased within the MHHs from 12 to 15% between 2010 and 2020. The figure implies that male heads potentially change their attention to vocational training while their counterparts pursue general education.

**Table 1:** Descriptive statistics of the data in 2010 and 2020.

Variables	Mean		Min		Max	
	2010	2020	2010	2020	2010	2020
*All sample*						
“With education” (general education)	0.79	0.85	0	0	1	1
+ Household head has a primary school certificate	0.28	0.26	0	0	1	1
+ Household head has a secondary school certificate	0.29	0.31	0	0	1	1
+ Household head has a high school certificate	0.15	0.19	0	0	1	1
+ Household head has a university or post-graduate degree	0.07	0.09	0	0	1	1
“With education” (vocational training)	0.13	0.14	0	0	1	1
+ Household head has a primary vocational training certificate	0.05	0.06	0	0	1	1
+ Household head has an intermediate vocational training certificate	0.07	0.06	0	0	1	1
+ Household head has an advanced vocational training certificate	0.001	0.02	0	0	1	1
The household head is a female	0.24	0.25	0	0	1	1
Single households	0.03	0.03	0	0	1	1
Married households	0.84	0.81	0	0	1	1
Widowed households	0.11	0.12	0	0	1	1
Separated or divorced households	0.03	0.04	0	0	1	1
Head's age	47.90	50.82	11	17	98	99
Total assets (million VND)	460.51	1294.33	0	0.3	30,717	35,065
Debt (million VND)	1.65	37.95	0	0	180	9150
Net worth (million VND)	458.85	1256.37	−28.13	−3925	30,717	31,557
Head's wage (million VND)	12.24	39.26	0	0	533	870
Other members' wage	15.85	53.73	0	0	465	798
Other income sources	97.08	217.42	0	0	16,897	34,699
Household size (equivalent scale)	3.04	2.85	1	1	9.25	8.73
Living in an urban area	0.29	0.33	0	0	1	1
“Kinh” ethnicity	0.85	0.85	0	0	1	1
Number of observations	8758	8554				
*Male-headed households*						
“With education” (general education)	0.82	0.87	0	0	1	1
“With education” (vocational training)	0.12	0.15	0	0	1	1
Total assets (million VND)	423.97	1266.70	0	0.3	22,102	31,557
Debt (million VND)	1.79	41.24	0	0	180	9150
Net worth (million VND)	422.19	1225.46	−16.19	−3925	22,102	31,557
Number of observations	6691	6390				
*Female-headed households*						
“With education” (general education)	0.72	0.79	0	0	1	1
“With education” (vocational training)	0.13	0.11	0	0	1	1
Total assets (million VND)	578.76	1375.89	0	1.4	30,717	35,065
Debt (million VND)	1.21	28.24	0	0	70	5050
Net worth (million VND)	577.55	1347.65	−28.13	−417	30,717	31,475
Number of observations	2067	2164				

In addition to changes in educational achievements between FHHs and MHHs, a household's wealth, the difference between a household's total assets and debts, is also different between FHHs and MHHs. Notably, while there was an approximately threefold increase in MHHs' wealth from VND 422 million VND in 2010 to more than VND 1.2 billion in 2020, FHHs performed less effectively at around 2.3 times during this period of ten years, from VND 577 million to more than VND 1.3 billion. Better wealth accumulation in MHHs narrows the gaps between them and their counterparts from 37 to 10% in 2010 and 2020, respectively. However, regardless of these improvements within MHHs, from the poorest and the wealthiest households, we observe different changes between MHHs and FHHs.

In particular, the poorest household in MHHs appears to have worsened from a negative net worth of 16.2 million VND in 2010 to 3.9 billion VND in 2020. The poorest households in FHHs reveal a better economic change in their household's financial security, from net debt of 28.1 million VND in 2010 to 417 million VND in 2020. Specifically, in 2010, the poorest household in MHHs (whose net worth is negative 16.2 million VND) had a better situation than those in the FHHs (whose net worth is negative 28.1 million VND). Inversely, in 2020, the situation changed, and the poorest household in MHHs suffered a substantial loss of wealth at nearly negative four billion VND, while the poorest household in FHHs faced a relatively moderate reduction to approximately negative 417 million VND. At the top of wealth distribution, an MHH overtakes its counterparts as the wealthiest household in 2020 with a small difference, such as 31.5 billion VND of net worth in MHH compared to 31.4 billion VND in FHH. However, generally, because of a broad spread between the lowest and highest value of the net worth in MHHs, the wealth distribution within MHHs reveals a wider dispersion than that within the FHHs.

Following [16], we employ quantile regression to observe the different effects of education and gender on household wealth across the wealth distributions. This approach provides us with insights into the simultaneous effect of education and gender on household wealth at different wealth levels. Besides, using the PSM method, we ensure the robustness of our results as we can overcome the endogenous problem of selection biases arising in the survey data.

## 4 Empirical results

Table 2 (for general education) and Table 3 (for vocational training) present empirical findings on the impacts of education and gender of the households' heads and their joint effect on household wealth in 2020 and 2010. Table 2 presents the empirical results on the effect of education and gender of the households' heads and their joint effects on household wealth in the Vietnamese context in the most recent decade, from 2010 to 2020. Education in this table is proxied by general education. Our findings indicate that the education of the households' heads contributed to increased household wealth in Vietnam in 2010 and 2020. However, the effects are more significant in 2020 than in 2010, consistent with previous studies [17], suggesting a trend of increasing returns to education over time. This could indicate that the knowledge-based economy is becoming more prevalent, and individuals with higher educational levels are better positioned to take advantage of the economic opportunities. In addition, interestingly, the effect of education of the households' heads on household wealth in 2010 increased from the lower to the higher quantile of household wealth in Vietnam. This result suggests that investments in education can lead to improved income-earning opportunities and financial stability. Education is again considered important for low-income households to improve their economic circumstances. However, this effect decreased from a lower to a high quantile of household wealth in 2020, implying that education may contribute to dealing with wealth inequality. The household heads with higher educational levels from low-income households may experience greater wealth accumulation, potentially reducing income disparities with other households. Our findings also indicate that FHHs have higher wealth accumulation at the lower quantiles. Interestingly, this effect was observed in 2020 but not in 2010, a signal of reducing gender inequality in income and wealth in Vietnam.

**Table 2:** The effects of education proxied by general education and gender of the households’ heads on the household wealth in Vietnam, 2020 versus 2010 surveys using a quantile regression with a matching sample.

Variables	25th percentile		50th percentile		75th percentile	
	2020	2010	2020	2010	2020	2010
Has a primary school certificate	2.394***	1.133***	1.878***	1.229***	1.640***	1.133***
	(0.146)	(0.105)	(0.103)	(0.089)	(0.100)	(0.101)
Has a secondary school certificate	2.239***	1.480***	1.824***	1.535***	1.565***	1.611***
	(0.119)	(0.092)	(0.084)	(0.078)	(0.082)	(0.089)
Has a high school certificate	2.248***	1.472***	1.832***	1.499***	1.462***	1.370***
	(0.110)	(0.098)	(0.078)	(0.083)	(0.075)	(0.095)
Has a university or post-graduate degree	2.371***	1.684***	1.939***	1.771***	1.677***	1.710***
	(0.119)	(0.109)	(0.084)	(0.092)	(0.081)	(0.105)
Head's gender (0: male; 1: female)	0.225*	−0.066	0.201**	−0.043	0.103	−0.047
	(0.115)	(0.100)	(0.082)	(0.085)	(0.079)	(0.097)
Head's gender* Has a primary school certificate	0.463	−0.622**	0.340	0.411*	0.495*	0.370
	(0.397)	(0.275)	(0.280)	(0.233)	(0.271)	(0.265)
Head's gender* Has a secondary school certificate	0.663**	0.280	0.629***	0.383**	0.532***	0.073
	(0.296)	(0.227)	(0.209)	(0.193)	(0.203)	(0.220)
Head's gender* Has a high school certificate	0.625***	0.110	0.533***	0.172	0.443***	0.092
	(0.226)	(0.181)	(0.160)	(0.153)	(0.154)	(0.174)
Head's gender* Has a university or post-graduate degree	0.517**	0.301*	0.303**	0.253*	0.090	0.113
	(0.206)	(0.179)	(0.146)	(0.151)	(0.141)	(0.173)
Head's age	0.028***	0.023***	0.024***	0.024***	0.021***	0.023***
	(0.003)	(0.002)	(0.002)	(0.002)	(0.002)	(0.002)
Married households	2.132***	0.744***	0.451***	0.278**	0.019	−0.137
	(0.160)	(0.132)	(0.113)	(0.112)	(0.110)	(0.128)
Widowed households	2.158***	0.610***	0.412***	0.065	−0.096	−0.308**
	(0.189)	(0.157)	(0.133)	(0.133)	(0.129)	(0.151)
Divorced households	2.205***	0.927***	0.640***	0.567***	0.406**	0.431*
	(0.248)	(0.232)	(0.175)	(0.197)	(0.170)	(0.224)
Separated households	1.729***	0.794**	0.472*	0.478*	0.387	0.009
	(0.380)	(0.323)	(0.269)	(0.273)	(0.260)	(0.312)
Head's wage income (log)	−0.022***	−0.011	−0.008	−0.016***	−0.012**	−0.013**
	(0.007)	(0.007)	(0.005)	(0.006)	(0.005)	(0.007)
Other members' wage income (log)	−0.007	−0.004	−0.012***	−0.006	−0.011***	−0.003
	(0.006)	(0.005)	(0.004)	(0.005)	(0.004)	(0.005)
Other income sources (log)	0.213***	0.361***	0.218***	0.311***	0.120***	0.292***
	(0.021)	(0.019)	(0.015)	(0.016)	(0.014)	(0.018)
Household size (equivalent scale)	0.128***	0.166***	0.097***	0.138***	0.138***	0.088***
	(0.037)	(0.028)	(0.026)	(0.024)	(0.025)	(0.027)
Living in an urban area	0.150**	0.561***	0.251***	0.664***	0.341***	0.683***
	(0.069)	(0.058)	(0.049)	(0.049)	(0.047)	(0.056)
Belong to the “Kinh” ethnicity	0.629***	0.482***	0.524***	0.475***	0.580***	0.606***
	(0.096)	(0.078)	(0.068)	(0.066)	(0.066)	(0.075)
Constant	5.276***	4.170***	7.957***	5.844***	10.111***	7.153***
	(0.267)	(0.217)	(0.189)	(0.184)	(0.183)	(0.210)
Observations	2530	3612	2530	3612	2530	3612

**Table 3:** The effects of education proxied by vocational training and gender of the households’ heads on the household wealth in Vietnam, 2020 versus 2010 surveys using a quantile regression with a matching sample.

Variables	25th percentile		50th percentile		75th percentile	
	2020	2010	2020	2010	2020	2010
Has a primary vocational training certificate	0.173*	0.342***	0.189***	0.319***	0.059	0.198**
	(0.098)	(0.097)	(0.066)	(0.083)	(0.073)	(0.084)
Has an intermediate vocational training certificate	0.108	0.346***	0.101	0.211***	−0.043	0.155*
	(0.105)	(0.094)	(0.071)	(0.081)	(0.079)	(0.081)
Has an advanced vocational training certificate	0.258*	0.129	0.196*	0.081	0.094	0.032
	(0.152)	(0.438)	(0.102)	(0.376)	(0.114)	(0.377)
Head's gender (0: male; 1: female)	0.021	−0.205*	−0.101	−0.292***	−0.071	−0.116
	(0.133)	(0.122)	(0.090)	(0.105)	(0.099)	(0.105)
Head's gender * a primary training certificate	0.290	−0.113	−0.060	−0.258	0.113	−0.084
	(0.261)	(0.221)	(0.176)	(0.190)	(0.195)	(0.190)
Head's gender * an intermediate vocational training certificate	0.049	0.002	−0.195	0.039	−0.088	0.039
	(0.218)	(0.180)	(0.147)	(0.155)	(0.163)	(0.155)
Head's gender * an advanced vocational training certificate	−0.231	0.640	−0.163	0.980	−0.028	0.407
	(0.289)	(0.905)	(0.195)	(0.777)	(0.216)	(0.778)
Head's age	0.021***	0.012***	0.014***	0.012***	0.015***	0.011***
	(0.003)	(0.003)	(0.002)	(0.002)	(0.002)	(0.002)
Married households	1.216***	0.743***	0.156	−0.111	−0.012	−0.208
	(0.212)	(0.185)	(0.143)	(0.159)	(0.158)	(0.159)
Widowed households	0.997***	0.679***	0.021	−0.145	−0.260	−0.227
	(0.254)	(0.215)	(0.172)	(0.185)	(0.190)	(0.185)
Divorced households	1.103***	0.364	0.357*	−0.340	0.221	−0.128
	(0.287)	(0.317)	(0.193)	(0.272)	(0.214)	(0.273)
Separated households	0.765	0.758	−0.170	0.118	0.492	−0.193
	(0.603)	(0.622)	(0.406)	(0.534)	(0.450)	(0.535)
Head's wage income (log)	0.028***	0.038***	0.025***	0.036***	0.016***	0.037***
	(0.007)	(0.008)	(0.005)	(0.007)	(0.006)	(0.007)
Other members' wage income (log)	−0.001	−0.017**	0.001	−0.006	−0.003	0.003
	(0.007)	(0.008)	(0.005)	(0.006)	(0.005)	(0.006)
Other income sources (log)	0.319***	0.402***	0.303***	0.404***	0.236***	0.354***
	(0.026)	(0.026)	(0.017)	(0.023)	(0.019)	(0.023)
Household size (equivalent scale)	0.171***	0.103**	0.112***	0.034	0.122***	0.020
	(0.044)	(0.042)	(0.030)	(0.036)	(0.033)	(0.036)
Living in an urban area	0.519***	0.816***	0.626***	0.895***	0.697***	0.993***
	(0.072)	(0.070)	(0.049)	(0.060)	(0.054)	(0.061)
“Kinh” ethnicity	0.731***	0.526***	0.696***	0.554***	0.736***	0.527***
	(0.125)	(0.118)	(0.084)	(0.102)	(0.093)	(0.102)
Constant	5.509***	4.653***	7.966***	6.302***	9.491***	7.614***
	(0.344)	(0.323)	(0.232)	(0.278)	(0.257)	(0.278)
Observations	2404	2196	2404	2196	2404	2196

We now focus on the joint effect of education and gender of the household heads on household wealth in Vietnam. Our empirical results present positive and significant estimated coefficients of this interaction term across different quantiles of household wealth. Particularly, the interaction provides the largest effect when the female heads own the secondary diploma in 2020 (for all quantiles). Achieving a higher education leads to better economic conditions in FHHs in 2020, except for those in the top wealth distribution. These results in 2020 confirm a better return of education to household wealth in FHHs compared to a decade before in 2010. Particularly, in 2010, education had not improved the economic circumstances of FHHs compared to their counterparts. At the bottom of the wealth distribution, receiving primary school certificates in FHHs results in a significantly lower net worth for those with no educational certificate. However, in 2010, owning secondary or primary school certificates in FHHs improved the household's wealth in the middle distribution. On the other hand, with a university degree or post-graduate level, the household economic circumstances can only be improved when women were the household heads in 2010 at lower quantiles of the wealth distribution. The effects of education in FHHs on wealth accumulation in 2020 compared to 2010 imply that being a female head with a degree/certificate from a general education will increase household wealth. Policymakers should consider gender-inclusive education policies that provide equal access to quality general education for females.

Table 3 presents the empirical results when the education of the households' heads is proxied by a vocational training certificate. We observe that vocational training generally matters for household wealth across different quantiles, as confirmed in previous studies [[16], [17], [18], [19]]. However, our findings indicate that the effects are more significant in 2010 than in 2020. Besides, in 2010, the intermediate level of vocational training had the largest effect on wealth accumulation, and the effect was reduced toward upper quantiles. Meanwhile, in 2020, the advanced level of vocational training reveals the largest return to household wealth, especially in the lowest quantile. These findings align with those from Ref. [17], confirming that the effect of vocational training certificates was not significant or even negative (for those owning elementary vocational training certificates) in 2008. However, it changed to be significantly positive in 2018 (for intermediate or advanced training levels). In addition, the reduction in the return of vocational training to household wealth in the upper quantiles is also consistent with the findings by Ref. [17]. Interestingly, FHHs reveal lower wealth accumulation than their counterparts in 2010 and lower quantiles. The effect cannot be confirmed in 2020 and at the higher quantile of wealth distribution in 2010. Our results presented in Table 3 also indicate that the joint effect of education and gender of the households' heads on household wealth cannot be established in both 2010 and 2020 across various quantiles of household wealth in Vietnam.

Our findings from this empirical analysis align with those reported in previous studies on the role of Confucianism on gender inequality in Vietnam [[55], [56], [57], [58]]. Among these studies [55], concluded that Confucianism negatively affects gender inequality in labour, health, and educational outcomes. However, their findings also indicate that when females pursue more years of schooling, they perform better than males [56]. examined gender role attitudes in Vietnam using a sample of 8288 respondents. Their findings indicate that gender attitudes are affected by many determinants, including age, education, and others. Findings from Ref. [58] study confirmed that Confucianism in Vietnam has affected gender inequality since 1975.

## 5 Conclusions and policy implications

The Constitution of the Socialist Republic of Vietnam (1992), Article 35 emphasizes the importance of developing education as the nation's primary policy objective. Vietnam has made remarkable strides in advancing its education sector through several reforms. Over the past decade since 2010, there has been a notable surge of 125% in higher education enrollments. Efforts have been made to enhance the quality of teaching, and by 2020, higher education lecturers in Vietnam will be required to hold master's or doctoral degrees. The literacy rate is approximately 95.4% [59]. Remarkable reforms are also underway in the secondary-high-school-education systems, particularly regarding graduation examinations and university entrances.

Our study investigates the independent effect of education and gender of the households' heads and their joint effect on household wealth in Vietnam between 2010 and 2020. Data from the Vietnam Household Living Standards Surveys (VHLSS) are used for our analysis. In this study, education is proxied by either (i) a general education, including twelve-year schooling, university, and post-graduate education, or (ii) vocational training, including elementary, intermediate, and advanced training. Our analysis employs threshold regression and propensity score matching techniques to ensure the robustness of our empirical findings.

Key findings from our analysis can be summarized as follows. *First*, the household's heads' education (proxied as general education) supports household wealth accumulation in Vietnam in 2010 and 2020. However, these effects are more significant in 2020 than in 2010. We also find that the effects are more significant at the higher quantiles of household wealth than at the lower quantiles. *Second,* our empirical findings reveal that households with females as the household heads accumulated lower wealth in the lower quantiles of wealth in 2010 when vocational training was proxied for education. However, when general education is considered, FHHs perform better in wealth accumulation in 2020, especially at the lower wealth quantiles. *Third,* we find that when females are the household heads with general education degrees or certificates, household wealth will be higher in 2020. This effect cannot be confirmed in 2010. Our empirical findings are largely consistent across different settings.

Based on these results, education plays a key role in shaping Vietnamese society now and in the future. As such, the Vietnamese government should continue investing in education. Education has always been at the forefront of socioeconomic policies in Vietnam. Building an appropriate education system and promoting the acquisition of general education and training among Vietnamese people are vital for Vietnam's economic development. It is crucial for households with lower levels of wealth in both 2010 and 2020 to obtain intermediate vocational training to enhance their economic status. The cost of education in Vietnam has significantly increased. Increased tuition provides a solid basis for universities to be financially independent to deliver high-quality programs to students. However, increased tuition has become a real financial burden for Vietnamese households, particularly in rural regions and disadvantaged socio-economical areas. As such, lowering tuition fees should be considered to make education more accessible, particularly for economically disadvantaged students. Valuable lessons about sponsorship for tuition fees can be learned from Northern European countries, which are among the world's wealthiest and have well-regarded education systems. In these countries, tuition fees are minimal or nonexistent, and the government assumes responsibility for funding schools, maintaining adequate facilities, and ensuring high educational standards.

Gender bias is real, and it still exists in modern Vietnamese society. Policies targeting this fundamental issue are expected to achieve a more inclusive society in Vietnam. The National Strategy on Gender Equality for the 2021–2030 period has been adopted in Vietnam to enhance and support gender equality and women entrepreneurship in the Vietnamese context. Vietnam has committed to ensuring gender equality by establishing specific goals to promote and support gender equality as stipulated in Vietnam's Sustainable Development Goals (SDGs) by the United Nations. In achieving these important goals, the Vietnamese governments at all levels and their agencies should consider formulating and implementing various policies in setting the foundations to reduce gender inequality regarding pay and work opportunities for women and girls. Policies targeting encouraging women to minimise time spent on housework and increase education and skills improvement to be ready for work are appropriate and effective. Finally, in the current deep-rooted gender stereotypes and a 'gendered structure' economy, gender and gender equality should be considered to be integrated into the formal curriculums at schools and universities to ensure that both girls and boys are aware of the current circumstances regarding gendered-based inequality in pay, income, and work opportunities and the way forward in the country to ensure a more inclusive society with sustainable economic growth and inclusive social transformation.

Our study exhibits limitations which should be considered in future studies. The cross-sectional data used in this study prevents us from examining the long-term effects of the return on education in wealth accumulation in Vietnamese households. As such, further studies can use panel data analysis to consider the overall effects of education returns on household wealth accumulation. Besides, in our current research, the data limitation prevents us from investigating different kinds of vocational training based on the cost of learning and specific categories. Future research can be conducted to close this research gap. In addition, it would be the case that males still pay spousal support to their ex-wives if the female is separated or divorced. As such, the family's wealth accumulation may be affected. Data on the spousal support from the VHLSS surveys are not available. However, it will be an interesting issue to be considered in future studies when such data becomes available.

## Additional information

No additional information is available for this paper.

## CRediT authorship contribution statement

**Duc Hong Vo:** Writing – review & editing, Writing – original draft, Visualization, Validation, Supervision, Software, Resources, Project administration, Methodology, Investigation, Funding acquisition, Formal analysis, Data curation, Conceptualization. **Anh The Vo:** Writing – review & editing, Writing – original draft, Visualization, Validation, Software, Resources, Project administration, Methodology, Investigation, Formal analysis, Data curation, Conceptualization. **Chi Minh Ho:** Writing – review & editing, Writing – original draft, Visualization, Validation, Software, Resources, Project administration, Methodology, Investigation, Formal analysis, Data curation, Conceptualization.

## Declaration of competing interest

The authors declare the following financial interests/personal relationships which may be considered as potential competing interests: Duc Hong Vo reports that this research is funded by Ho Chi Minh City Open University under grant number E2021.02.2.
